# ICES: Data, Discovery, Better Health

**DOI:** 10.23889/ijpds.v4i2.1135

**Published:** 2020-03-11

**Authors:** MJ Schull, M Azimaee, M Marra, RG Cartagena, MJ Vermeulen, M Ho, A Guttmann

**Affiliations:** 1 ICES, 2075 Bayview Avenue, G106, Toronto, ON, Canada (Authors are listed in the order of the sections)

## Background

ICES was founded in 1992 to study the health care system in Ontario and promote effective, efficient and equitable health care. Over 27 years later, the goal remains largely unchanged, though the institute has grown in size and impact. Originally known as the Institute for Clinical Evaluative Sciences, ICES was created as an independent not-for-profit research institute and given what was, at the time, unprecedented access to administrative health data records for the population of Ontario. ICES’ initial focus was to better understand the delivery of hospital services and translate its findings into better health care and policy. From modest beginnings with a handful of researchers located in a few hospital offices, ICES has grown to encompass a community of almost 500 scientists and staff across a network of seven physical sites in Ontario. The original focus on hospital-based services has expanded significantly and now includes research and analysis of community-based health services, health policy, Indigenous health, social determinants of health, and data science.

## Population Setting

ICES data covers the 13.4 million people living in Ontario, Canada’s largest province. When Ontarians receive health services outside of Ontario (in other Canadian provinces and territories and in the United States), ICES also receives records related to those services. Records of health services provided to non-Ontarians in the province of Ontario are also captured in the main health data at ICES; however, these records are not linkable to other data in most cases.

## Operating Model

ICES data are available in a single data repository that currently contains about 90 different data holdings.

## Data Linkage

The Ontario Health Insurance Plan (OHIP) is the universal health insurance system that provides almost all Ontario residents with health care services free at the point of delivery based on an OHIP card and its unique 10-digit identifier. This card must be presented each time a patient receives a health service that is covered under OHIP, such as a physician visit or a surgical procedure [1]. The OHIP cards are issued for newborns, immigrants and others who make Ontario their long-term residence. Health card numbers are retired as people die or relocate to another province or country, or reissued when persons return to Ontario after being away for long periods of time [1]. Some health services are not universal and are paid for by OHIP based on eligibility criteria, such as prescription drugs which are publicly funded for those under the age of 25 (if not covered by a private plan), those over the age of 64, living in a long-term care home or receiving social assistance.

The Registered Persons Database (RPDB) includes information on every individual who has been ever issued an OHIP card. RPDB captures almost all of Ontario’s 13.4 million residents. Raw data updates are provided to ICES by the Ontario Ministry of Health and Long-Term Care (MOHLTC) under a specific data sharing agreement. The RPDB file contains individual health card number, as well as demographics and personally identifiable information (e.g., surname, given names, sex, date of birth, earliest date of coverage, last date of contact with the health care system and residential postal code) [2].

The RPDB forms the spine for ICES record linkage and is updated on a monthly basis. Using a highly confidential and secure proprietary algorithm, each OHIP number in RPDB and any other health data with OHIP number is uniquely converted to an anonymous ICES Identifier. The unique Identifiers are used to combine information from different data holdings in the repository [1].

OHIP numbers are not collected or provided for every data holding available at ICES, especially in vital statistics and non-health data such as immigration databases. These data sources are linked deterministically and probabilistically with the RPDB using direct personal identifiers other than OHIP numbers (i.e., surname, given names, sex and date of birth). The spine-based record linkage model at ICES follows the Fellegi-Sunter method [3]. Currently ICES uses Automatch software for performing probabilistic record linkage [4]. In this process, surnames are standardized by implementing the New York State Identification and Intelligence System (NYSIIS) [5] phonetic conversion.

In order to optimize the scanning process of possible matched pairs, a technique called blocking is implemented during the probabilistic linkage process. This method partitions both files (the RPDB and the incoming data) into mutually exclusive and exhaustive subsets [2]. This allows the linkage to be performed within each block and therefore only records within the same block can be linked [6]. The possible matches (also known as grey area) are examined manually by data linkage experts. The manual resolution of grey area is a very resource and time intensive process. ICES is currently evaluating other record linkage techniques and tools to minimize the burden of this step and make record linkage more efficient.

## Data Sources

The ICES data repository consists of about 90 record-level data holdings comprising 1407 data tables. It encompasses much of the publicly funded administrative health services records for the Ontario population eligible for universal health coverage since 1986 in addition to registries and surveys. Other record-level datasets (including primary data collected by researchers such as clinical trial data) are integrated with repository data for specific projects. Currently, the repository’s health service and non-health data contain 20 billion records, more than 500,000 data elements and 1016 data points. Major categories of datasets held at ICES are listed in Table 1. Novel datasets starting to be linked to ICES administrative data include genomic data [7]. ICES does not currently routinely collect data on services delivered in purely private healthcare settings, those paid through private insurance or out-of-pocket payments.

**Table 1: Major Categories and Key Examples of Linkable Datasets Held at ICES*:** *See ICES Data Dictionary for more details https://www.ices.on.ca/Data-and-Privacy/ICES-data/Data-dictionary †Limited data and/or not available at population level ‡Ontario portion of national surveys and some provincial surveys on community health, health behaviours, and health care experience some of which are linkable to other ICES data §Population-level cohorts of chronic disease based on validated algorithms developed at ICES and updated annually

Dataset Category	Key Examples
Health Services	In-patient hospital records (from 1988) Emergency department and ambulatory care (from 2000) Physician billings (from 1991) Electronic medical records† (from 2010) Homecare (from 1994) Laboratory test results (from 2007) Mental health and addiction services (from 2005) Same day surgery (from 1991) Long-term care, complex care and rehabilitation services (from 1996) Publicly funded prescriptions and all narcotics/controlled substances (from 1990)
Acquired Cohorts and Registries	Cancer (from 1990) Bariatric surgery (from 2010) HIV (from 1995) Perinatal (from 2006) Organ donation (from 1995)
Care Providers	Physician demographics, specialty and other training information (from 1992) Physician practice payment model (from 2005)
Coding and Geography	Drug, diagnosis and geography conversion and reference tables
Facilities	Information on health service institutions (from 1988)
Financial	Cost of health care services (from 1992)
Population and Demography	Vital statistics (deaths only) (from 1990) Refugee and immigration status (from 1985) Derived ethnicity data (algorithm-based) (from 1990) Census profiles (from 1991) Population estimates and projections (from 1981)
Surveys‡	Canadian Community Health Survey (from 2001) Ontario Health Study (from 2009) Health Care Experience Survey (from 2006)
ICES-Derived Cohorts§	Asthma (from 1993) Cardiac (from 1991) Congestive heart failure (from 1991) Dementia (from 1996) Diabetes (from 1991) Inflammatory bowel disease (from 1991) Mother-baby pairs (from 1988) Patient-physician-hospital referral networks (from 2005) Rheumatoid arthritis (from 1993)
Non-Health Sector Data	Environmental† Transportation† Education† Government disability support

## Architecture and Information Technology

The ICES primary technology infrastructure is called the Research Analytic Environment (RAE), and it provides analytic tools such as Python, SAS, Stata and R; other software is added as needs arise. The systems use a mix of RedHat Linux and Windows servers on an Active Directory domain. The backend of the RAE consists of Virtual Machines (VMs) using Cisco UCS blades and NetApp All-Flash Storage managed with VMWare. This provides the flexibility and performance to scale resources based on demands.

The RAE is contained in a secure, isolated network at ICES Central. Other ICES sites access this environment through remote sessions across a private network. The ICES data repository consists of SAS datasets and Microsoft SQL databases that are secured to ensure that RAE users only access data that they are authorized to read. Currently the RAE houses over 5 TB of data, uses over 80 CPU cores, and over 1.2 TB of RAM to support a user base of over 250 analytic staff, trainees and scientists.

Two additional environments exist at ICES: 1) The ICES Data and Analytic Virtual Environment (IDAVE) provides remote secure access for external researchers and third party projects. Each IDAVE user accesses a research cluster preloaded with analytical software tools along with specific directories and resources the user is authorized to access. No data or documentation can be copied or transferred from the IDAVE without undergoing re-identification risk assessment by ICES staff; 2) The Health Artificial Intelligence Data and Analysis Platform (HAIDAP) is a high-performance computing cluster in a private cloud environment, which is an extension of the RAE, built to accommodate research projects that require greater computational resources and specialized software packages for artificial intelligence, machine learning and natural language processing. Each user is capable of loading additional software from an approved catalog based on their profile. Access to all analytic environments at ICES is controlled through credentialing and two-form authentication.

## Governance, Legislation, Funding and Management

ICES is a not-for-profit corporation under Ontario’s Corporations Act [8] and a registered charity. ICES is designated as a Prescribed Entity under Ontario’s Personal Health Information Protection Act, 2014 (PHIPA) [9]. This designation permits a Health Information Custodian [10] (e.g., a hospital or physician practice) to provide Personal Health Information [11] to ICES for the purpose of analysis or compiling statistical information with respect to the management, evaluation or monitoring of health services, including allocation of resources and planning [12]. Patient consent is not required for this disclosure of PHI to occur.

There is a strict regulatory framework in place under PHIPA [13], which requires that ICES has in place practices and procedures to protect the privacy of the individuals whose Personal Health Information (PHI) it receives and to maintain the confidentiality of the information, and these practices and procedures must be reviewed and approved by Ontario’s Information and Privacy Commissioner of Ontario (IPC) every three years. This includes ICES privacy and security policies and procedures, templates, logs, human resources and other organizational practices. There are other laws that are relevant with respect to the ability of ICES to collect and use data from other sources, including federal privacy legislation [14], provincial freedom of information legislation as well as Ministry-specific enabling legislation. All relevant statutes need to be analyzed before ICES can collect and use data.

ICES has worked closely for several years with First Nations and Métis partners to develop specific data governance and sharing agreements for Indigenous-driven analyses using administrative health data and registries [15]. Each data governance agreement is guided by principles of Indigenous sovereignty. The agreements now govern access to the Indian Registry, which identifies status First Nations individuals in Ontario (about 200,000 people), and the registry of the Métis Nation of Ontario. ICES works with Indigenous partners to apply a decolonized lens and, using Indigenous models of well-being, works with communities to build internal research capacity by training Indigenous researchers.

Since its inception, ICES has received funding from the Ontario MOHLTC to maintain its data repository to support health system stakeholder needs. In 2014, ICES received additional funding from the MOHLTC and the Canadian Institutes for Health Research (CIHR) Strategy for Patient-Oriented Research (SPOR) to expand data and analytic access to third party researchers. The additional costs of investigator- and other stakeholder-driven research projects are borne by grants and contracts.

## Consent Model

As a Prescribed Entity under PHIPA, ICES is able to collect individual-level personal health information without patient consent from a variety of health stakeholders (entitled Health Information Custodians in PHIPA) and a limited number of non-health stakeholders. Through its recently created Public Advisory Council, ICES consults with members of the public to understand public perspectives on the use of health information for research via the Prescribed Entity model, and ensure that their values are considered in key decisions around its use.

## Privacy by Design

Only a restricted group of ICES staff have permission to handle fully-identifiable data for the purposes of de-sensitising, i.e. creating the anonymous ICES Identifier, removing direct personal identifiers, and carrying out data quality and destruction procedures. These individuals serve in a highly trusted position, receive special training and sign robust confidentiality agreements. Access to data within ICES is subject to an individual’s assigned data access level, based on role and need such that the access is limited to the minimum amount of data and in the least identifiable form which is required to serve the purpose identified. ICES’ role-based access model is described below [13].

## Data Access

ICES currently provides two types of data and analytic services: internal access for projects conducted by ICES-affiliated scientists and trainees (ICES Projects) and external access for projects conducted by third-party researchers (ICES Data and Analytic Services Projects).

## ICES Projects (Internal)

When ICES appoints a scientist, fellow or student they become an agent of ICES and may access data for the purposes of analysis to support evaluation, planning and monitoring of the health system without research ethics board approval. All ICES agents receive privacy and security training and sign confidentiality agreements annually. Each ICES project triggers a privacy impact assessment (PIA) conducted by the ICES Privacy and Legal Office to identify and minimize any privacy risks in the project, as well to ensure that the data request is permissible under PHIPA, ICES policies and procedures or any contractual obligations with data partners. The PIA reviews project objectives and the requested datasets, including the rationale for each requested data holding. Each project team member with access to ICES research outputs (summary data) must be named on the project PIA and those who are not ICES agents sign a non-disclosure agreement. ICES researchers are only able to access data after it has been de-sensitised and has been determined to be necessary for the purposes identified.

ICES analytic staff have direct access to the data repository and typically link and prepare project datasets as well as conducting analyses for most projects through role-based access. ICES trainees have access to datasets that have been prepared and further de-sensitised by ICES staff. Access to the data repository is contingent on the completion of a dataset creation plan that outlines in detail how the data will be used to complete the project objectives. The responsible ICES scientist and ICES analytic staff further attest that the DCP is consistent with the objectives approved in the project PIA. Median time from submission of the project PIA to data access by the ICES research team is 50 days.

Research outputs may be shared with project team members listed on the project PIA and ICES staff listed on the DCP. Prior to release of summary data outside of the project team, the responsible ICES scientist must conduct a re-identification risk assessment and ensure it is documented. Re-identification risk assessment comprises suppression of small cells and confirming that the summary data could not foreseeably be used (either alone or with other information) to re-identify an individual.

Project teams that require linkage of external data with data in the ICES repository may request that ICES collect project-specific data on their behalf. For publicly available data and other types of data that do not include personally identifiable or personal health information (PII or PHI), a data sharing request form is completed by ICES’ Privacy & Legal department to ensure adherence to the terms of use. Data that include PII or PHI require that a Data Sharing Agreement (DSA) be established between ICES and the data custodian. In all cases, data integration is conducted by ICES staff. 

## Third-Party Projects (External)

ICES Data & Analytic Services (DAS) enables data accessibility to investigators external to ICES. DAS provides access to risk-reduced (highly desensitized) data sets created from ICES’ data holdings; analytic support; and complete data analysis and report-writing services. DAS also imports researcher-collected data for linkage with ICES data holdings. Access to risk-reduced data sets is currently not available to private sector investigators. Private sector investigators are only eligible for complete data analysis performed by ICES, resulting in reports that contain summary data.

Publically funded researchers may perform their own analyses and create reports using risk-reduced data sets accessed through IDAVE. Multifactor authentication is required to access IDAVE, and external peripherals connected to the user’s local computer are disabled. Access is further restricted to directories and resources the users are authorized to access. No data or documentation can be copied or transferred from IDAVE without undergoing re-identification risk assessment by ICES staff as per ICES polices.

To access DAS services, the researcher submits a project request form, available on the ICES website. DAS assesses eligibility and project feasibility, followed by a consultation to review project objectives and timelines, and DAS issues a Confirmation of Feasibility (COF). As part of the project request form and COF, which becomes part of a DAS agreement, source of research funding as well as the roles and affiliations of the researchers, are confirmed as this directly impacts eligibility for remote access to risk-reduced data sets. The researcher seeks research ethics board approval for the project and satisfies any other conditions that must be met prior to project initiation, as specified in the COF. The ICES Privacy & Legal department reviews the submission to the research ethics board and corresponding approval. A DAS agreement must be signed, outlining terms and conditions for provision of services and estimated cost. For projects requesting the importation of external data to ICES, a data sharing agreement will be negotiated as part of the DAS Agreement. The researcher and members of their project team sign confidentiality agreements, designating them as Authorized Researchers, and may now access their customized research-ready data on the IDAVE. The median time for data set availability to the external researcher after signing the Services Agreement is 37 days.

When the project team needs to remove output files (aggregate data only) from IDAVE, DAS assesses requested files for re-identification risk, and vetted files are emailed to the researcher. Upon project completion, the researcher informs DAS of project closure and access to the IDAVE is terminated.

## Noteworthy Outputs

**Figure 1: ICES Productivity 2003/04 to 2018/19:**
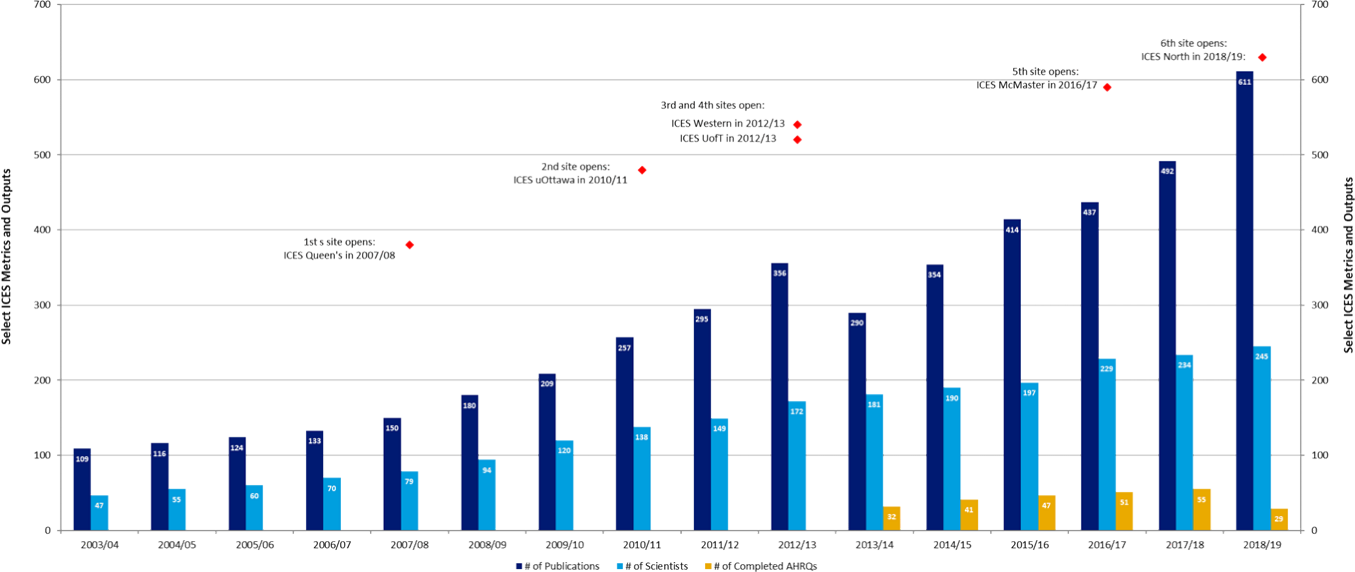


**Figure 2: Examples of ICES Research Impact Stories*:**
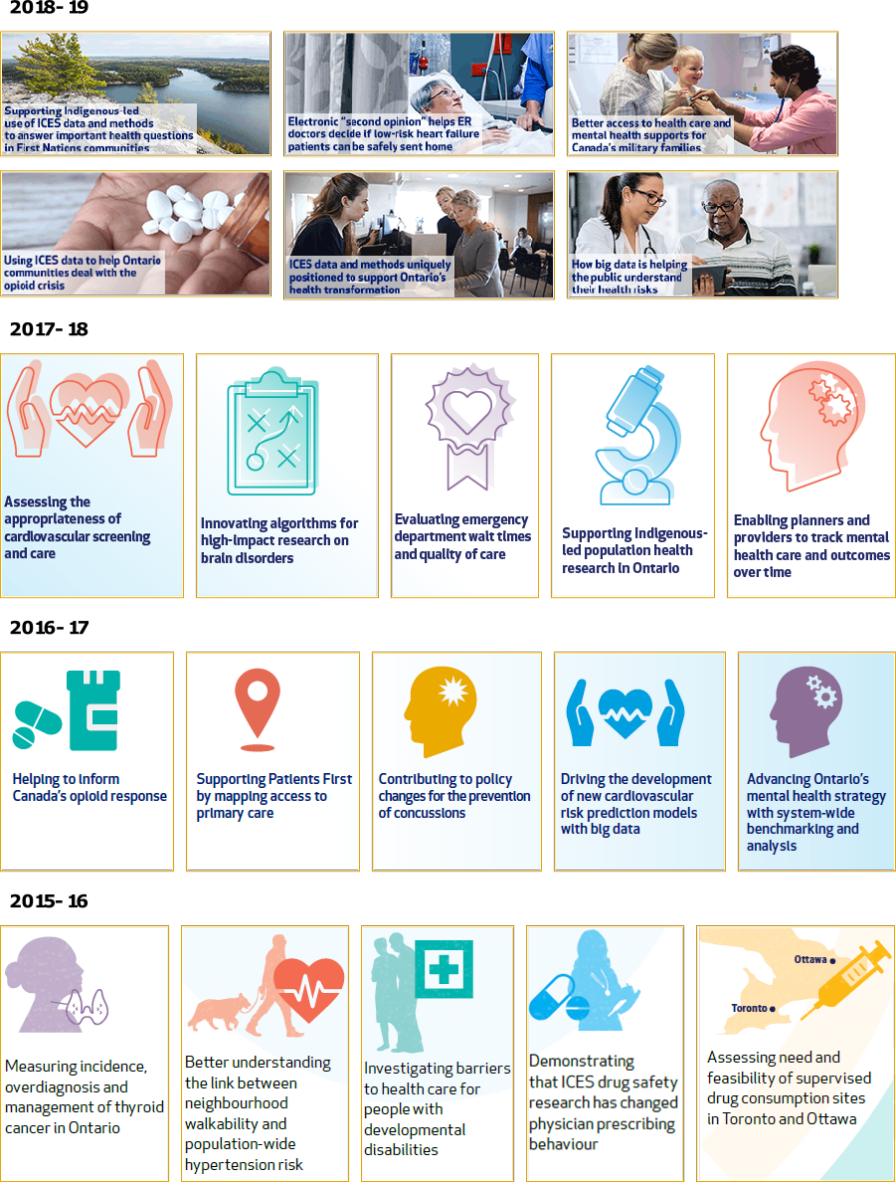
*See https://www.ices.on.ca/Newsroom/Impact-Stories

The growth of ICES capacity has resulted notable increases in output over time (Figure 1), with more than 2200 peer-reviewed publications by ICES scientists in the last five years. During that same period, ICES published an additional 238 reports and briefs under the Ministry’s Applied Health Research Question (AHRQ) program, in which ICES produces analyses and reports in direct response to requests from public sector health system stakeholders. These studies and reports have had measurable impact on clinical practice, research methods, legislation, policy and programs (Figure 2). For example, an innovative risk calculator [16] underpins the living kidney donor international clinical practice guideline [17] and has led to changes in donor criteria and increased numbers of eligible donors. A series of rapid-response ICES studies [18,19] provided evidence that a new policy on high-dose opioid prescribing would not have a negative impact on palliative care patients. Work to define informal physician referral and hospital relationships and indicators of integrated care [20] are now being used to inform significant health system reorganization. ICES research has been notably considered as part of legislative changes that have included bans on the use of cellphones while driving, strengthened legislation around return to sport after concussions, and greater restrictions on firearms currently being considered by the Government of Canada.

About 30% of ICES publications are led by scientists who are based at one of six smaller sites located across Ontario, the most recent of which opened in northern Ontario in 2018. The majority of ICES studies include an ICES staff member as a co-author, reflecting the depth of their involvement on those projects. Researchers external to ICES have made more than 500 requests for data access on IDAVE since its launch in 2014, with more than 90% of the requests being feasible. Some have gone on to result in publication in high-impact journals [21].

## Discussion

ICES’ longevity as a stand-alone independent research institute reflects a number of factors. First was the view that it would be more than a data repository, and would focus on attracting and retaining a strong and multi-disciplinary mix of researchers and highly qualified staff committed to secure data management and high quality evaluation and research. A second factor was the development of strong links to health system decision and policy makers, that helped to ensure that ICES would be able to address high priority questions from these stakeholders. A third factor was to ensure that ICES enabled investigator-driven research along with responsiveness to stakeholder priorities to help increase overall impact.

ICES has also grown by modernising data access models. Whereas originally access to data was limited to those physically onsite at its central Toronto location, ICES began establishing the first of its six additional sites in 2006 (Figure 1). This enabled researchers outside Toronto to access the ICES central data repository, though they still required an ICES appointment. With additional funding in 2014 from the Ontario MOHLTC and the Canadian Institutes of Health Research (CIHR) Strategy for Patient-Oriented Research (SPOR), ICES created the IDAVE platform to offer secure remote data access and analytic support to any researcher in Canada.

ICES’ evolution included growth in research programs and priorities as well. ICES added several research programs over time, including primary care and population health, renal disease, and mental health and addictions. ICES has also launched several institutional priority initiatives. For example, ICES has been working closely with First Nations, Inuit and Métis partners for several years to develop a number of unique partnerships that have enabled Indigenous-driven analyses using ICES data. In October 2017, ICES formalized an Indigenous Portfolio at ICES with dedicated staff, a scientist network and a scientific lead, and has begun to recruit additional Indigenous researchers and trainees. Another institutional priority initiative is focused on data science and Big Data, and a report [22] led by ICES scientists laid out a road map for engagement in this evolving area of science, such as the launch of high-performance computing capacity at ICES and partnerships with AI/ML research institutes.

On the national scene, ICES has worked closely with similar data institutes, centres and data platforms across Canada to address jurisdictional barriers and better enable multi-province research. This culminated with funding from the CIHR to launch, in 2019, the SPOR Canadian Data Platform, a distributed data network created by Health Data Research Network Canada [23] and involving all provinces and one territory in Canada [24]. The platform promises to be a game changer for national and multi-province health research in Canada, and similarly offers the potential for international collaborations without the need for data to cross jurisdictional boundaries.

Challenges also exist, such as ever-present funding constraints, though ICES has been fortunate to receive continuous support from the Ontario government. In particular, this has helped to fund core infrastructure and personnel necessary to maintain the data platform, but which generally cannot be recovered from research grants and contracts. Finally, in Canada as in other countries, substantial public debate has been generated around access to and use of data, including questionable practices in the private sector access as well as oversight of research [25]. ICES has begun to work with members of the general public, through mechanisms like focus groups and by establishing a standing Public Advisory Council, in order to engage with and involve members of the public to ensure that data-intensive health research is trustworthy and within the bounds of social licence. 

## Conclusion

Over almost three decades, ICES has evolved and grown in size, outputs and impact. It has increased its national and international linkages and collaborations. Yet the institute’s focus has remained to use health and social data to generate trusted research and evidence that can make policy better, improve care, and strengthen the health care system.

## Ethics statement

Ethics approval was not sought for this manuscript as its sole purpose is to describe ICES’ population data centre.
